# Neuregulin1β improves cognitive dysfunction and up-regulates expression of p-ERK1/2 in rats with chronic omethoate poisoning

**DOI:** 10.1186/s12993-014-0050-8

**Published:** 2015-02-07

**Authors:** Lixia Rong, Kun Ding, Meizeng Zhang, Yunliang Guo

**Affiliations:** Institute of Cerebrovascular Diseases, Affiliated Hospital of Qingdao University, Qingdao, 266003 China; Department of Neurology, Affiliated Hospital of Qingdao University, Qingdao, 266003 China

**Keywords:** NRG1β, Chronic, Omethoate, Cognitive dysfunction, p-ERK1/2

## Abstract

**Objective:**

To observe the effects of neuregulin1β (NRG1β) on the level of phosphorylated ERK1/2 (p-ERK1/2), and explore the therapeutic mechanism of NRG1β on the cognitive dysfunction in rats with chronic omethoate poisoning.

**Methods:**

Rats with strong learning and memory ability, 50 in total, were selected by Y-electric maze test. Among which, 15 rats were randomly selected into control group, and the rest 35 rats were used to establish experimental cognitive impairment models by being injected with omethoate subcutaneously. The 30 cases of successful cognitive impairment models were randomly divided into model group and treated group consisting of 15 rats, respectively. Then rats in treated group were injected with NRG1β into their lateral ventricles, while rats in control and model groups were given equal volume of PBS simultaneously. The cognitive capacity of rats was evaluated with Y-electric maze. The morphology and ultrastructure of hippocampus were observed by hematoxylin eosin (HE) staining and transmission electron microscopy (TEM) respectively. The expression of p-ERK1/2 was determined by immunohistochemical (IHC) staining and Western blotting.

**Results:**

Compared with rats in model group, the cognitive ability of rats with omethoate exposed (model and treated groups) reduced significantly, along with the obvious damage of hippocampal neurons and the expression of p-ERK1/2 decreased significantly (*P* < 0.05). And after treatment with NRG1β, the cognitive activity of treated rats was improved obviously, and the injury of hippocampal neurons was milder and the expression of p-ERK1/2 increased significantly more than those in model rats (*P* < 0.05).

**Conclusion:**

In chronic omethoate poisoning rats, NRG1β can promote the phosphorylation level of ERK1/2 in hippocampal neurons, and play an important role in the improvement of cognitive function.

## Introduction

As a kind of economical and efficient pesticides, organophosphorus pesticides (OPs) have been widely applied around the world. With strong toxicity, the remaining OPs can be accumulated for a long time after entering the body, and would be greatly harmful to human health [1]. Epidemiological data indicated that long-term exposure to low dose OPs would lead to cognitive dysfunction, mainly shown as the decline of learning and memory ability [1,2]. Up to now, no effective treatment has been found for the cognitive dysfunction caused by chronic poisoning with OPs. Previous studies [3,4] showed that chronic poisoning with OPs could cause the reduction of muscarinic acetylcholine receptors(mAChRs) in brain, as well as its affinity for acetylcholine(Ach), finally leading to the tolerance to Ach. As one of the most important neurotransmitters in the central nervous system, Ach is widely distributed in brain, and involved in the neuronal activity of hippocampus and cortex. It can regulate the synaptic plasticity and plays an important role in the processes of learning and memory. Rosenblum et al. [5] found that the realization of learning and memory function which is mediated by acetylcholine neurons depends on the activation of ERK1/2 signaling pathway. Recently, one study demonstrated that in chronic OPs poisoning rats, the learning and memory ability and the phosphorylated activation level of ERK1/2 in brain were reduced significantly [6]. So it was speculated that the cognitive dysfunction caused by OPs might be related with the blocking of ERK1/2 signal transduction pathway. As a member of epidermal growth factor family, neuregulins (NRGs) can activate multiple signal transduction pathways (including ERK1/2 pathway) after binding to its ErbB receptors, and display a number of roles in the developing and mature nervous systems. Several lines of evidence [7,8] suggest that in rats with cerebral ischemia-reperfusion injury, NRG1β played an important role in anti-apoptosis effect by regulating the expression of STAT3, GFAP and AQP-4. Subsequently, some studies found NRG1β could also inhibit inflammation and apoptosis in brain of dementia rats [9,10], as well as prevent the damage of long-term potentiation (LTP) in hippocampus and regulate the synapse formation and plasticity [11]. Therefore, in animals with chronic organophosphorus poisoning, the protective effects of NRG1β on hippocampal neurons and its improvement on the cognitive impairment can be expected, as well as it effects on the activation of ERK1/2.

Omethoate is one of the most frequently used organophosphorus pesticides in China. It’s an structural analog of dimethoate with strong toxicity. Due to its high toxicity, frequent use and appearance, omethoate is of public concern and on the list of Priority Monitoring Pesticides published by the Ministry of Environmental Protection of the People’s Republic of China. In this study, the authors aimed at establishing chronic organophosphate poisoning models by injecting omethoate subcutaneously, and exploring the possible mechanism of NRG1β improving cognitive dysfunction by determining the phosphorylation level of ERK1/2 (p-ERK1/2) in hippocampus.

## Materials and methods

### Animals

Total of 50 healthy adult male *Wistar* rats (weighting 230-250 g, SPF grade) were provided by Experimental Animal Center of Qingdao Drug Inspection Institute (SCXK (LU) 20120010). Animals were housed at the ambient temperature of 21-25°C, with natural illumination and free access to food and water for one week to adapt the environment. This experiment was approved by the Ethics Committee of Qingdao University Medical College (QUMC 2011-09). The local legislation for ethics of experiment on animals and guidelines for the care and use of laboratory animals were followed in all animal procedures.

### Animal screening

After acclimatizating in Y-electric maze for 3 minutes, each rat was placed in one arm of the Y-electric maze and stimulated by electricity (60 V, 0.5 ~ 0.7 mA, 5 s delayed), at the same time one of the other two arms was given light as a signal of safety, and the other arm without light signal was dangerous with electrical current flowing. If the rat ran directly to the safe area in 10 s, we considered it as a correct response, otherwise, error response. After the rat escaped into the safe area, the light continued lasting for 30 seconds for the rat to consolidate memories, then the light was turned off and the second training started from the arm where the rat currently stayed (that is the safe area of last training), and either arm was given light signal. Carry on again and again thus ten times every day for 7 days. And in the seventh day, during the ten continuous trainings, the rats reacted correctly for at least 9 times (50 in total) were taken into the experiment. 15 rats were selected randomly into control group, and the rest 35 were used to establish chronic omethoate poisoning cognitive impairment models.

### Establishment of models

The 35 rats were subcutaneously injected with 5 mg/kg (2 ml/kg) omethoate (Shandong Dacheng Pesticide Co. Ltd., CAS No: 1113-02-6, purity: 40%) every day for 4 weeks; and the 15 rats in control group (n = 15) were given equal volume of normal saline simultaneously. After the exposure for 4 weeks, the learning and memory abilities of rats were tested by Y-electric maze for 10 times continuously. If one rat reacted correctly less than 6 out of 10 times, we considered the rat had cognitive impairment. The 30 successful model rats of cognitive impairment were randomly divided into model group (n = 15) and treated group (n = 15).

### Interventions

NRG1β (Cat. 100-03, Pepro. Tech. Co. Ltd. USA) was dissolved and diluted with 0.1 mol/L PBS buffer solution to 1 g/L (1 μg/μl). On the first and the eighth day after modeled successfully, each rat in treated group was injected with 5 μl of NRG1β through the left and the right lateral ventricle (AP = 0.8 mm, ML/MR = 1.1 mm, DV = 3.6 mm) respectively. And the rats in control group and model group were given equal volume of PBS buffer solution simultaneously. During the intervention period, 2 rats in model group died. After exposed to omethoate for 4 weeks, the immunity of exposed rats was damaged seriously. The two exposed rats with no treatment may be too weak to survey.

### Memory reappearance test

Seven days later after the second administration of NRG1β, all rats (43 in total, with 15 in both control group and treated group, 13 in model group) were tested by Y-electric maze for 10 times continuously.

### Specimen collection

#### Paraffin section

Five rats in control group, 5 in treated group and 4 in model group were randomly selected and anesthetized with 10% chloral hydrate (3 ml/kg) intraperitoneal injection. Then 200 ml normal saline and 200 ml 4% paraformaldehyde solution were perfused into the heart successively. After that, the brain slice from 1 mm to 6 mm behind optic chiasm was cut down and fixed in 4% paraformaldehyde solution for 24 hours. Then the brain slices were dehydrated with graded series of ethanol, cleared with xylene and embedded in paraffin in turn. Then the embedded tissue was cut into sections of 5 μm thickness by a microtome and adhered to the glass slices processed with poly-lysine and stored at 4°C.

#### Ultrathin sections

Five rats in control group, 5 in treated group and 4 in model group were randomly selected and anesthetized with 10% chloral hydrate. Then the whole brain was taken out, and bilateral hippocampi were stripped out. Small amount of fresh tissue was cut from bilateral hippocampi into some small pieces of about 1 mm × 1 mm × 1 mm, and fixed in 2.5% glutaraldehyde for 24 hours. And then followed with 0.1 mol/L phosphate buffer rinsed for 15 min × 3 times; fixed in 1% osmium tetroxide for 2 hours, 0.1 mol/L phosphate buffer rinsed for 15 min × 3 times, and then dehydrated with gradient of acetone, embedded in Epon812 epoxy resin. Finally, the embedded blocks were cut by the ultramicrotome (Leica EM UC6, Germany) into ultrathin sections of 50 nm thickness which were placed on the nets prepared with polyvinyl formal and stored at 4°C.

#### Protein extraction

Five rats were randomly selected from each group and anesthetized with 10% chloral hydrate. And the whole brain was taken out with bilateral hippocampi stripped out on the ice. Then 100 mg hippocampus tissue was placed into a 1.5 ml EP tube with a right amount of cell lysis buffer (1 ml lysis buffer +10 μl PMSF, No. P0013B, Beyotime Institute of Biotechnology). Then the tissue was grinded fully and homogenized by ultrasonic and placed on ice for one hour. Then the homogenate was centrifuged with 12000 rpm for 15 minutes at 4°C (Eppendorf 5417R, Germany). The supernate was collected to determine the concentration of protein by BCA Protein Assay Kit (No. P0010, Beyotime Institute of Biotechnology), and finally saved at -80°C.

### Detection indexes

#### Hematoxylin-eosin (HE) staining

The paraffin sections were conventionally dewaxed and rehydrated, followed with staining in hematoxylin for 5 minutes, color separation with 1% hydrochloric acid alcohol for 5 seconds, and staining in eosin for 2 minutes. After that the paraffin sections were dehydrated by graded series of ethanol, cleared by xylene, and finally sealed with neutral gum. The structure of hippocampus was observed under microscope (Leica DMI4000B, Germany) at 400 times magnification. Four non-overlapping horizons were randomly selected from each slice for observation. The denatured cell index (DCI = the number of denatured cells/the number of total cells) was analyzed to evaluate the injury severity of hippocampus.

#### Transmission electron microscopy (TEM)

The 3% uranyl acetate-alcohol saturated solution (pH = 3.5) and 6% lead citrate dye liquor (pH = 12) were prepared in advance. A drop of 3% uranyl acetate-alcohol saturated solution was dripped in a petri dish with clean wax-scale, then covered by the nets with ultrathin sections which contacted with the dye liquor to stain for 30 min, rinsed with double-distilled water for 10 min × 3 times, sucked up the water, covered with the 6% lead citrate dye liquor which was dripped in another dish with clean wax-scale, and the sections were contacted with the dye liquor to stain for 5 min, rinsed with non-carbon dioxide double-distilled water for 10 min × 3 times, blotted up water and dried at room temperature. The ultrastructure of neurons was observed under the transmission electron microscope (JEM-1200EX, Japan).

#### Immunohistochemical (IHC) staining

Rabbit anti-rat p-ERK1/2 antibody and Rabbit anti-rat ERK1/2 antibody were provided by Cell signaling technology Co. Ltd. PV-6001 immunohistochemisty kit and DAB chromogenic solution were provided by Beijing Golden Bridge Biotech. Co. Ltd. The paraffin sections were conventionally dewaxed and rehydrated, and operated according to the specification of PV-6001 kit, colored by DAB and re-dyed with hematoxylin. Finally the paraffin sections were conventionally dehydrated by graded series of ethanol, cleared by xylene, and sealed with neutral gum. The positive cells showed brown granules in cytoplasm under light microscope. Four non-overlapping horizons were selected randomly from each slice under microscope at 400 times magnification. And LEICA Qwin image-processing system was applied to analyze the mean optical density (OD) of each horizon.

#### Western blotting

The objective proteins p-ERK1/2 (44, 42 kD), ERK1/2 (44, 42 kD) and β-action (42KD) were separated by sodium dodecyl sulfate polyacrylamide gel electrophoresis (5% concentrated gel on 80 V and 10% separating gel on 120 V successively) and transferred onto the polyvinylidene fluoride (PVDF) membranes (90 min with 360 mA). Then the PVDF membrane was blocked in 5% skimmed milk solution for 2 h at room temperature. After that the PVDF membrane was incubated with p-ERK1/2 primary antibody (1:2000, CST, #4370) at 4°C overnight and horseradish-peroxidase tagged goat anti-rabbit secondary antibody (1:10000, Abcam, ab136817) for 1 h at room temperature successively. After each step, the PVDF membrane must be rinsed sufficiently to avoid nonspecific coloration. The gel film images was developed in A-B mixed developing agent and scanned with Biospectrum 810 Imaging System. Then the PVDF membrane was dealt with stripping buffer to remove the antibodies. After that the membrane was blocked with skimmed milk solution again, then re-incubated with ERK1/2 primary antibody (1:1000, CST, #9102) or β-actin primary antibody (1:200, Wuhan Boster Biological Co. Ltd., BA2305), and secondary antibody. Finally the images of ERK1/2 and β-actin were developed. And the gray value of all protein bands were measured and analysed with the Quantity one software (Bio-Rad, USA). The relative gray value of protein was calculated by the ratio of p-ERK1/2 or ERK1/2 gray value to β-actin gray value.

### Statistical analysis

SPSS 17.0 software was applied for the statistical analysis. The data was expressed as mean ± standard error $\left(\overline{x}\pm s\right)$. The analysis of variance and *t-*test were applied for the two-group comparison.

## Results

### Test of memory reappearance ability

The correct reaction times of poisoning rats (model group and treated group) reduced significantly than that in control rats (*t* = 15.24, 7.69, *P* < 0.05), but the correct times in treated rats were obviously more than that in rats of model group (*t* = 7.05, *P* < 0.05). Compared with model rats, the learning and memory abilities of rats in treated group were improved (Table 1).

**Table 1:** **The number of correct responses to electrical stimulation**

**Group**	**n**	**Before modeled**	**After modeled**	**After treated**
Control group	15	9.60 ± 0.51	8.60 ± 0.83	8.93 ± 0.70
Modeled group	13	9.46 ± 0.52	4.31 ± 0.85*	4.23 ± 0.93*
Treatment group	15	9.53 ± 0.52	4.27 ± 0.80*	6.67 ± 0.90*^#^

*Compared with control group, *P* < 0.05; ^#^Compared with modeled group, *P* < 0.05.

### HE staining

**Figure 1:**
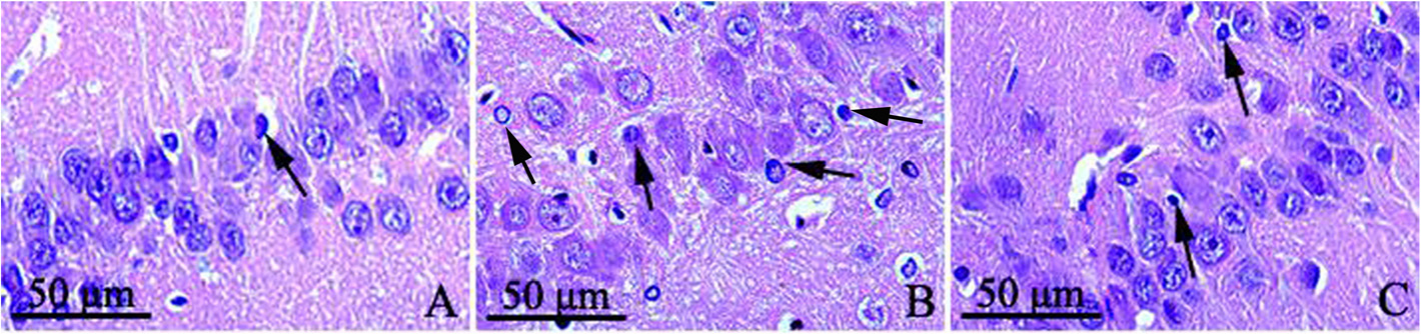
**The morphological structure of hippocampus, HE × 400. A**: Control group, **B**: Modeled group, **C**: Treated group (Denatured cell was pointed by black arrow). Hippocampal neurons in control rats were arranged in alignment with nucleolus obviously; In model group, neurons were loosely arranged,with cytoplasm condensed and nucleus pycnosis. In treated group, with neurons arranged closer, the damage was slighter than that in model rats.

The structure of hippocampal neurons in control rats was normal, and cells arranged in alignment, stained into shallow blue purple, and nucleolus obviously; In model group, there was a certain degree of damage in hippocampal with neurons loosely arranged, neuron shrinker, cytoplasm condensed, nucleus pycnosis, and cells dyed darkness. In treated group, with neurons arranged closer, the damage of hippocampal neurons was slighter than that in rats of model group, and the number of denatured cells was also less than that in model group (Figure 1).

The analysis of denatured cell index (DCI): the DCI of hippocampus in poisoning rats (model group and treated group) was higher than that in control rats (*t* = 12.46, 9.38, *P* < 0.05); and the DCI of hippocampus in treated rats was obviously lower than that in rats of model group (*t* = 7.03, *P* < 0.05) (Table 2).

**Table 2:** **The analysis of denatured cell index (DCI) in HE staining**

**Group**	**n**	**DCI**
Control group	5	0.099 ± 0.019
Modeled group	4	0.236 ± 0.040*
Treatment group	5	0.158 ± 0.020*^#^

*Compared with control group, *P* < 0.05; ^#^Compared with model group, *P* < 0.05.

### Ultrastructure of hippocampus

**Figure 2:**
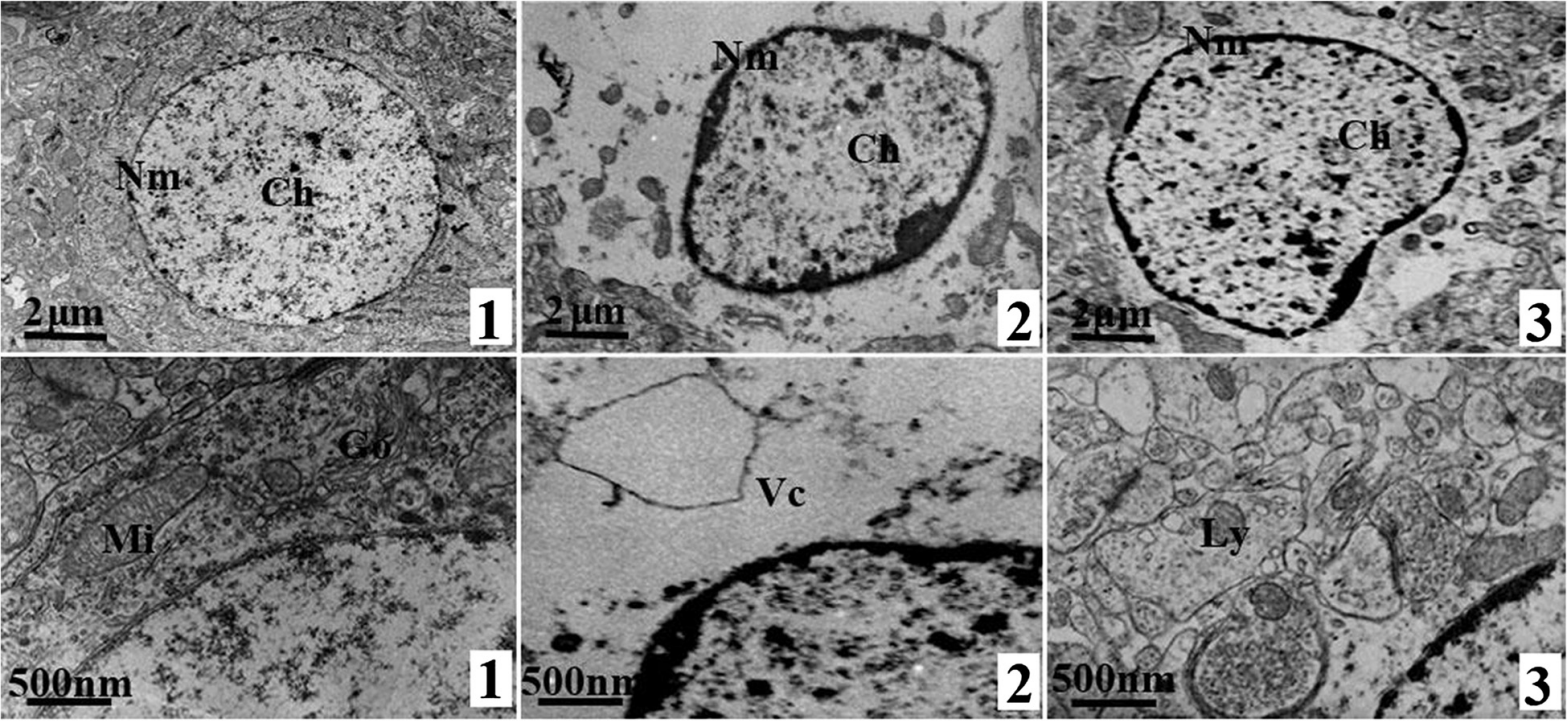
**Ultrastructure of neurons in hippocampus, TEM × 5000 (up) and × 20000 (down).** **1**: Control group, **2**: Modeled group, **3**: Treated group; Nm: Nuclear membrane, Ch: chromatin, Mi: Mitochondria, Go: Golgi apparatus, Vc: vacuoles caused by the dissolution of cytoplasm, Ly: Lysosome. In control group, neurons had distinct outline and cytoplasm with uniform chromatin; while the morphology of neurons in model group were irregular with uneven distribution of chromatin and dissolved cytoplasm vacuoles; the damage of neurons in treated group was milder than that in model group, and only some slightly gathered chromatin and a small amount of dissolved cytoplasm can be found.

In control group, the structure of neurons was regular with distinct outline and cytoplasm, and the chromatin was uniform; in model group, the morphology of neurons was irregular with uneven distribution of chromatin, and the vacuoles caused by the dissolution of cytoplasm can also be observed; the damage of neurons in treated group was milder than that in model group, and only some slightly gathered chromatin and a small amount of dissolved cytoplasm can be found (Figure 2).

### Immunohistochemical (IHC) staining

**Figure 3:**
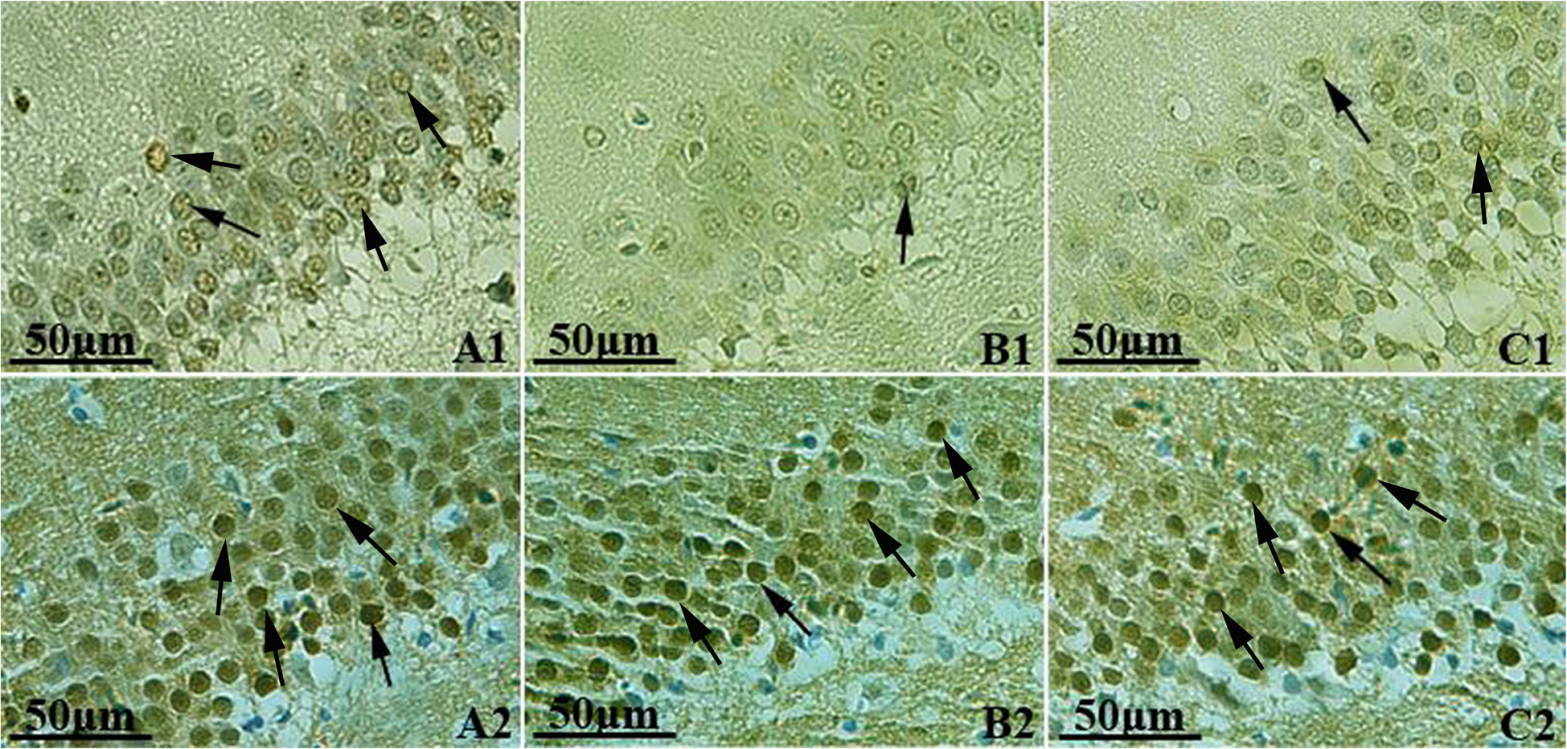
**The expressions of p-ERK1/2 and ERK1/2 in hippocampus, IHC Staining × 400. A**: Control group, **B**: Modeled group, **C**: Treated group; 1: p-ERK1/2, 2: ERK1/2 (Positive cells were pointed by black arrow). In control group, a large amount of p-ERK1/2 positive cells stained as brownish yellow could be observed. Compared with control group, neurons in model group were stained lightly and the number of p-ERK1/2 positive cells reduced significantly. In treated group, the number of p-ERK1/2 positive cells was more than that in model group. A large amount of ERK1/2 positive cells can be observed in each group with no significant difference.

In control group, hippocampal neurons were arranged in neat rows, and a large amount of p-ERK1/2 positive cells stained as brownish yellow could be observed under microscope. Compared with control group, hippocampal neurons in model group were arranged loosely and clutter with lighter staining. The number of p-ERK1/2 positive cells in model group reduced significantly, and the analysis of optical density (OD) showed that there was significant difference between model and control groups (*t* = 8.13, *P* < 0.05). In treated group, the number of p-ERK1/2 positive cells was more than that in model group, and analysis of OD showed a significant difference (*t* = 4.76, *P* < 0.05). Immunohistochemical staining of ERK1/2 showed that there were a large amount of ERK1/2 positive cells in each group, and the analysis of mean OD showed no significant difference (*t* = 0.69-1.50, *P* > 0.05) (Figure 3, Table 3).

**Table 3:** **Optical density of p-ERK1/2 and ERK1/2 IHC Staining in hippocampus**

**Group**	**n**	**p-ERK1/2**	**ERK1/2**
Control group	5	59.38 ± 10.86	96.56 ± 12.76
Model group	4	27.82 ± 12.42*	93.60 ± 13.01
Treatment group	5	47.03 ± 11.69*^#^	100.57 ± 14.48

*Compared with control group, *P* < 0.05; ^#^Compared with modeled group, *P* < 0.05.

### Western blotting

**Figure 4:**
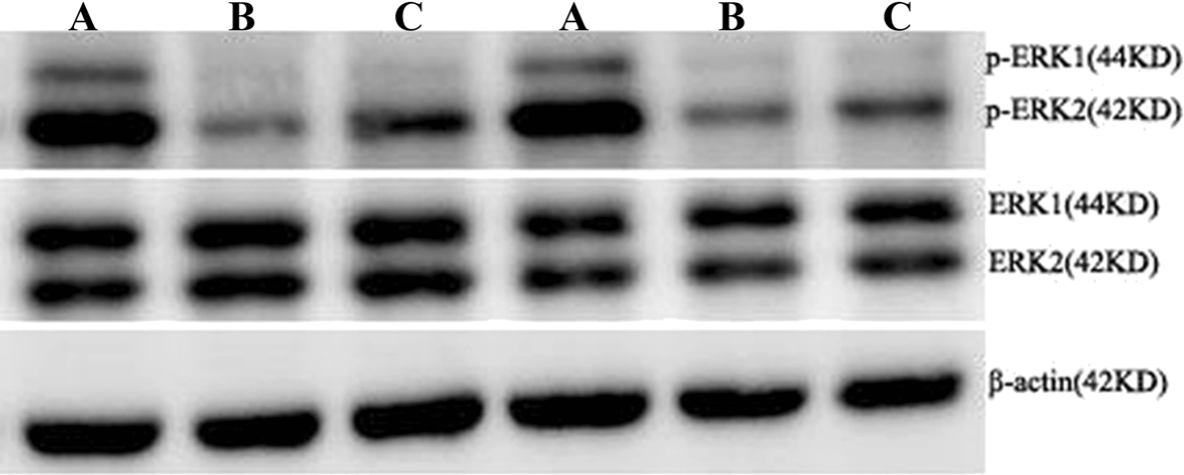
**The expressions of p-ERK1/2 and ERK1/2 in hippocampus. A**: Control group, **B**: Modeled group, **C**: Treated group. There was no significant difference in the expression of total ERK1/2 in hippocampus among control, model and treated group rats, while the expression of p-ERK1/2 in model and treated groups decreased significantly compared with control group. And in compare with model group, the expression of p-ERK1/2 in treated group increased significantly.

There was no significant difference in the expression of total ERK1/2 in hippocampus among control, model and treated group rats (*t* = 1.56, 0.97, *P* > 0.05), while the expression of p-ERK1/2 in model and treated groups decreased significantly and the gray value showed significant difference (*t* = 9.49, 6.97, *P* < 0.05) compared with control group. And in compare with model group, the expression of p-ERK1/2 in treated group increased significantly, and the analysis of gray value showed the difference was statistically significant (*t* = 3.65, *P* < 0.05) (Figure 4, Table 4).

**Table 4:** **The relative gray value of p-ERK1/2 and ERK1/2 in hippocampus (%)**

**Group**	**n**	**p-ERK/actin**	**ERK/actin**
Control group	5	22.5 ± 2.5	42.9 ± 2.8
Model group	5	9.4 ± 1.8*	40.3 ± 2.4
Treatment group	5	13.2 ± 1.6*^#^	40.0 ± 5.9

*Compared with control group, *P* < 0.05; #Compared with modeled group, *P* < 0.05.

## Discussion

The ERK signaling cascade is a central MAPK pathway that plays a role in the regulation of various cellular processes such as proliferation, differentiation, survival and migration [12,13]. The ERK pathway also has profound effects on the regulation of apoptosis by the post-translational phosphorylation of apoptotic regulatory molecules including Bad, caspase 9 and Bcl-2 [14,15]. The protective effect of ERK on neurons may also be related to the reduction of some inflammatory factors. In the brains of aged rats, the decline of activated ERK is often accompanied by the increase of IL-1β. In addition, the reduction of ERK can cause the decline of some neurotrophic factors, such as BDNF and NGF. In brief, NRG1β plays a role in neuroprotective effect by activating ERK1/2.

Besides, the abilities of learning and memory are also related to the activation of ERK1/2. The function of acetylcholine neurons about learning and memory depends on the activation of ERK1/2 signaling pathway. After acetylcholine binds with mAChRs, ERK1/2 pathway can be activated, thus regulating the ability of learning and memory [13]. After binding with cholinergic neurotransmitter, the activated receptor can activate G protein specifically and promote the Gα subunit with GDP-bound converting to GTP-bound. Then the affinity of Gα and Gβγ subunits decreases, which furtherly causes the separation of heterotrimer. The separated Gα (GTP) subunit and Gβγ subunit can activate some molecules, such as PKC, PI3K or Src, to activate ERK1/2 signaling pathway.

The activation of ERK1/2 signaling pathway is essential for various forms of synaptic plasticity. As an important form of synaptic plasticity, long-term potentiation (LTP) has been an important cellular model for the study of learning and memory. LTP in hippocampus is believed to be an important basis for learning and memory functions [16]. In 1997, when English and Sweat [17] used PD098059 to block the activation of ERK1/2, they found the induction of LTP in hippocampus was inhibited significantly, which confirmed the important role of ERK1/2 in the induction of LTP. After that, more and more studies confirmed that ERK1/2 signaling pathway played an important role in the formation of LTP and the processes of learning and memory [18-26]. The activation of ERK1/2 could in turn affect the activation of some nuclear transcription factors which were related to synaptic plasticity and the synthesis of related functional protein, thus inducing the formation of LTP. For example, activated ERK1/2 could phosphorylate the Ser133 site of cAMP response element-binding protein (CREB), thus leading to the activation of CREB and the transcription of cAMP response element (CRE)-related genes, finally inducing the formation of LTP [27]. As an important nuclear transcription factor, CREB was a necessary regulatory molecule in the formation of long-term memory. The activation of CREB played an important role in the maintenance of late LTP in hippocampus, and was involved in the process of learning and the consolidation of memory [28], and the inhibition of CREB in hippocampus could cause spatial memory impairment of rats [29].

In this study, after the exposure to omethoate for 4 weeks, the results of Y-electric maze showed that the cognitive capacity of model rats declined significantly compared with that of control rats. Under light microscope, the hippocampal neurons of model rats arranged loosely and clutter with cytoplasm condensed and nucleus pycnosis; under electric microscope, the morphology of hippocampal neurons was irregular with uneven distribution of chromatin, and the phenomenon of apoptosis, vacuolization and necrosis could also be observed. Compared with model group, the learning and memory abilities of rats in treated group were improved with milder damage of hippocampus, which indicating that NRG1β played a neuroprotective effect against the hippocampus injury and could improve the cognitive ability of rats with omethoate poisoning. The results of IHC staining and western blot showed that the phosphorylated activation level of ERK1/2 declined significantly in the hippocampus of poisoning rats, but the activation level of ERK1/2 in rats with NRG1β treated was obviously higher than that in rats of model group. This indicated that ERK1/2 signal transduction pathway was involved in the cognitive impairment caused by chronic omethoate poisoning, and NRG1β played a neuroprotective effect by activating the ERK1/2 signal pathway, so as to improve the cognitive function of rats. In future, we will furtherly explore the optimum dose of NRG1β on improving the cognitive impairment of rats caused by chronic omethoate poisoning and the therapeutic effect of NRG1β in other kinds of cognitive impairment.
